# Regulation of Oligomeric Organization of the Serotonin 5-Hydroxytryptamine 2C (5-HT_2C_) Receptor Observed by Spatial Intensity Distribution Analysis*

**DOI:** 10.1074/jbc.M115.644724

**Published:** 2015-03-30

**Authors:** Richard J. Ward, John D. Pediani, Antoine G. Godin, Graeme Milligan

**Affiliations:** From the ‡ Institute of Molecular, Cell and Systems Biology, University of Glasgow, Glasgow G12 8QQ, Scotland, United Kingdom,; the § University of Bordeaux, LP2N, UMR 5298, F-33405 Talence, France, and; the ¶ Institut d'Optique Graduate School and CNRS, LP2N, UMR 5298, F-33405 Talence, France

**Keywords:** seven-helix receptor, epidermal growth factor receptor (EGFR), G protein-coupled receptor (GPCR), serotonin, single-molecule biophysics

## Abstract

The questions of whether G protein-coupled receptors exist as monomers, dimers, and/or oligomers and if these species interconvert in a ligand-dependent manner are among the most contentious current issues in biology. When employing spatial intensity distribution analysis to laser scanning confocal microscope images of cells stably expressing either a plasma membrane-associated form of monomeric enhanced green fluorescent protein (eGFP) or a tandem version of this fluorophore, the eGFP tandem was identified as a dimer. Similar studies on cells stably expressing an eGFP-tagged form of the epidermal growth factor receptor demonstrated that, although largely a monomer in the basal state, this receptor rapidly became predominantly dimeric upon the addition of its ligand epidermal growth factor. In cells induced to express an eGFP-tagged form of the serotonin 5-hydroxytryptamine 2C (5-HT_2C_) receptor, global analysis of construct quantal brightness was consistent with the predominant form of the receptor being dimeric. However, detailed spatial intensity distribution analysis demonstrated the presence of multiple forms ranging from monomers to higher-order oligomers. Furthermore, treatment with chemically distinct 5-HT_2C_ receptor antagonists resulted in a time-dependent change in the quaternary organization to one in which there was a preponderance of receptor monomers. This antagonist-mediated effect was reversible, because washout of the ligand resulted in the regeneration of many of the oligomeric forms of the receptor.

## Teaser

**Background:** The quaternary organization of G protein-coupled receptors remains a subject of considerable debate.

**Results:** The serotonin 5-HT_2C_ receptor is a mixture of monomers, dimers, and higher-order oligomers. These become predominantly monomers upon antagonist treatment.

**Conclusion:** SpIDA indicates that the 5-HT_2C_ receptor exists as mixtures of forms that are regulated in an antagonist-dependent manner.

**Significance:** SpIDA delivers new insights into receptor quaternary structure.

## Introduction

Defining the quaternary organization of protein complexes without resorting to cellular disruption followed by biochemical analysis remains a major challenge. Resonance Energy Transfer (RET)^3^ techniques, including various forms of fluorescence resonance energy transfer (FRET), are well established approaches that can identify protein-protein interactions in intact cells (1–4). However, such approaches are generally unable to also identify co-expressed monomeric species. Moreover, although it is conceptually possible to discriminate between dimeric and higher-order complexes, it is often difficult, in practice, to discriminate between such forms in RET-based studies. Furthermore, with expression to high levels of proteins containing suitable fluorophores, RET signals that reflect crowding or random “bystander” effects are often difficult to resolve from true signals that reflect direct interactions between the proteins being studied. Conversely, the absence of RET signals does not necessarily mean the absence of protein-protein interaction and could simply reflect the incapacity of the constructs to transfer energy (*e.g.* large distance between or orientation of the fluorophores) or, similarly, the presence of another binding partner between the two studied proteins.

The structural organization of certain transmembrane receptor families (*e.g.* the broad group of single transmembrane domain receptor tyrosine kinases) is well established, as is the basic concept that ligand binding to many of these receptors results in their dimerization to promote signal transduction (5–7). However, for many other transmembrane receptor classes, there is substantially less clarity on these matters. Spatial intensity distribution analysis (SpIDA) directly measures fluorescent macromolecule densities and oligomerization states sampled within single images (8). The method is based on fitting intensity histograms calculated from images to obtain density maps of fluorescent molecules and their quantal brightness (QB) (9). SpIDA has recently begun to be employed to determine both basal organization and also changes in such organization in response to activation of a number of transmembrane receptors (10–11). Indeed, in some of the initial applications of SpIDA, the proportion of epidermal growth factor receptor (EGFR) present as dimer was shown to increase in response to the addition of the ligand EGF, whereas that of monomer decreased (8). In contrast, the single transmembrane domain axonal guidance receptor Robo-1 has been shown to be a constitutive dimer in the basal state, and this was unaffected by the addition of the ligand Slit 2 (12).

One of the most actively studied groups of transmembrane receptors is the seven-transmembrane domain G protein-coupled receptor (GPCR) family. This reflects both their preponderance in number and their targeting by a host of therapeutic medicines. Although it is well established that members of the small, class C or “glutamate-like” receptor grouping are constitutive dimers (13, 14) or possibly dimers of dimers (15), understanding of the quaternary organization of the much larger group of class A or “rhodopsin-like” receptors lags substantially behind (16). Although highly studied, this is one of the most contentious areas in current biology with very different conclusions being reached. These range from opinions that consider most of the receptor population to exist as monomer, with only random collisions suggesting quaternary structure (17–21), to others that indicate the vast majority, or even all, of the receptor exists as either dimers or higher-order oligomers (22–25). The implications of such organization for both novel drug design and understanding of the mode of action of current medicines (26, 27) are also an actively debated topic. Herein, we have employed SpIDA to address this question for the 5-HT_2C_ receptor, a class A GPCR that responds to the neurotransmitter serotonin. Prior to performing such studies, a series of control experiments were performed. First, enhanced GFP (eGFP) containing the A206K mutation that limits the tendency of this protein to self-associate (28) or a tandem of this fluorophore was attached to the plasma membrane of transfected cells by the addition of a myristoylation and palmitoylation consensus sequence (29). Analysis by SpIDA demonstrated that the A206K eGFP tandem was recorded as a dimer compared with the single molecule of A206K eGFP. Furthermore, over a broad range of expression levels, a single molecule of plasma membrane-associated A206K eGFP was reported consistently as monomeric. Subsequently, by linking A206K eGFP to the single transmembrane domain EGFR, the addition of the ligand EGF was shown to convert a large proportion of the receptor from monomer to dimer. When expanded to the 5-HT_2C_ receptor, this GPCR was shown to exist at the basolateral plasma membrane of cells stably expressing this receptor in forms ranging from monomer to higher-order oligomers. Treatment with a number of antagonists of the 5-HT_2C_ receptor (reversibly) reduced this complexity to favor monomeric and dimeric forms of the receptor. These results demonstrate that this receptor can exist in multiple forms, that interaction with ligands can alter this ensemble substantially, and that methods that report averages of receptor organizational structure fail to identify the level of complexity present.

## EXPERIMENTAL PROCEDURES

### 

#### 

##### Materials

General laboratory chemicals were from Sigma-Aldrich (Poole, UK) or Fisher Scientific (Leicester, UK). Otherwise, DNA restriction endonucleases, calf intestinal alkaline phosphatase, T4 DNA polymerase, and T4 ligase were from New England Biolabs (Hitchin, UK). The Wizard Plus SV Miniprep kit was from Promega (Southampton, UK). NuPage Novex precast 4–12% BisTris gels, NuPage MOPS SDS running buffer, and native polyacrylamide gels and their associated buffers, markers, and sample preparation reagents were from Invitrogen (Paisley, UK). The QIAfilter Plasmid Maxi Kit, PCR purification kit, and QIAquick gel extraction kit were from Qiagen (Crawley, UK). Agarose was from Flowgen Biosciences (Nottingham, UK). Secondary horseradish peroxidase-conjugated antibody was from Sigma-Aldrich. ECL reagent was purchased from Pierce (Tattenhall, UK). Polyethylenimine (PEI) was from Polysciences Inc. (Warrington, PA). [^3^H]Mesulergine was from PerkinElmer Life Sciences (Chalfont Road, UK). Serotonin hydrochloride (5-HT) was from Sigma-Aldrich. SB242084 (6-chloro-2,3-dihydro-5-methyl-*N*-[6-[(2-methyl-3-pyridinyl)oxy]-3-pyridinyl]-1*H*-indole-1-carboxyamide dihydrochloride), SB243213 (2,3-dihydro-5-methyl-*N*-[6-[(2-methyl-3-pyridinyl)oxy]-3-pyridinyl]-6-(trifluoromethyl)-1*H*-indole-1-carboxamidedihydrochloride), and RS102221 (8-[5-(2,4-dimethoxy-5-(4-trifluoromethylphenylsulfonamido)phenyl-5-oxopentyl]-1,3,8-triazaspiro[4.5]decane-2,4-dione hydrochloride) were from Tocris Bioscience (Abingdon, UK).

##### DNA Constructs

**FIGURE 1.**
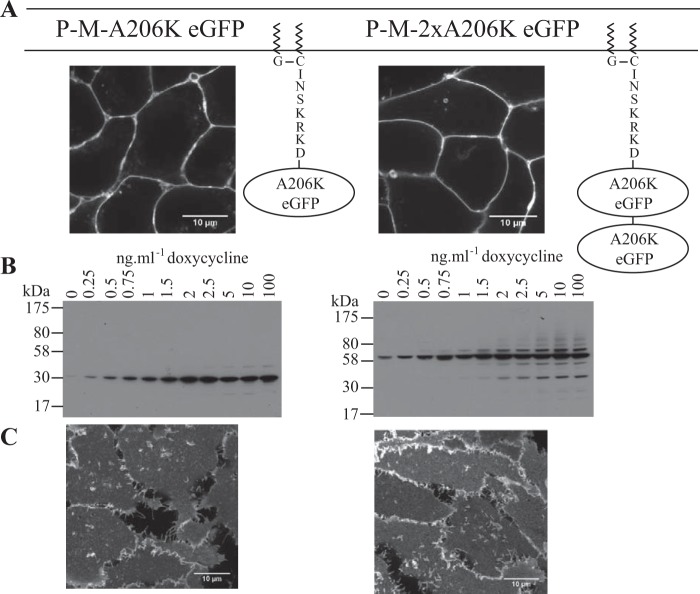
**Regulated expression patterns of palmitoylation-myristoylation-linked forms of A206K eGFP.** Flp-In^TM^ T-REx^TM^ 293 cell lines harboring either palmitoylation-myristoylation-linked A206K eGFP (P-M-A206K eGFP) (*A*, *left*) or an equivalent palmitoylation-myristoylation-linked form of the A206K eGFP tandem (P-M-2xA206K eGFP) (*A*, *right*) were induced to express these constructs by treatment with doxycycline (100 ng·ml^−1^, 24 h). Confocal images identify their plasma membrane delivery. *Scale bar*, 10 μm. *B*, lysates of cells that had been treated with the indicated concentration of doxycycline for 24 h were resolved by SDS-PAGE and immunoblotted with an anti-GFP antiserum. *C*, confocal images of the basolateral surface of such cells are shown. *Scale bar*, 10 μm.

Constructs expressing a single enhanced (1x) green fluorescent protein, tandem (2x) eGFPs, and the human 5-HT_2C_ receptor fused at its carboxyl terminus to eGFP (5-HT_2C_-eGFP) were a gift of Dr. K. Herrick-Davis (Albany, NY). These constructs were all based upon the vector pEGFPN1 (Clontech), and all had been modified to incorporate an A206K mutation in the eGFP to reduce any tendency for the fluorescent protein to homodimerize (28). In order to localize the 1x-A206K eGFP and 2x-A206K eGFP to the plasma membrane, a palmitoylation-myristoylation sequence was added to the amino terminus of the fluorescent protein(s) by subcloning the following oligonucleotides between the NheI and BglII sites of the pEGFPN-1 polylinker after annealing them together: oligo 1, CTAGCGCCACCATGGGATGCATCAACTCGAAGAGAAAGGATA; oligo 2, GATCTATCCTTTCTCTTCGAGTTGATGCATCCCATGGTGGCG. This added the following palmitoylation-myristoylation sequence to the amino terminus: Met-Gly-Cys-Ile-Asn-Ser-Lys-Arg-Lys-Asp (29) (Fig. 1*A*). The EGFR was fused to A206K eGFP at the carboxyl terminus by subcloning PCR-amplified EGFR into pEGFPN1 at AscI and NotI (which were added to the vector by inserting a suitable linker).

To make inducible Flp-In^TM^ T-REx^TM^ stable cell lines, the palmitoylation-myristoylation-A206K eGFP (P-M-A206K eGFP), palmitoylation-myristoylation-2xA206K eGFP (P-M-2xA206K eGFP), and 5-HT_2C_-A206K eGFP were excised with NheI and NotI and subcloned into the pcDNA5/FRT/TO vector at EcoRV-Not1 (after blunting the NheI site). The pcDNA5 EGFR-A206K eGFP construct was made by a similar strategy except that it had to be assembled as a three-way ligation due to the presence of a NotI site at the fusion junction. All constructs were verified by sequencing.

##### Cell Lines

All cells were maintained in a humidified incubator with 95% air and 5% CO_2_ at 37 °C. Human embryonic kidney cells (HEK293T) were grown as a monolayer in Dulbecco's modified Eagle's medium (DMEM) supplemented with 10% (v/v) fetal bovine serum, 2 mm l-glutamine, 100 units·ml^−1^ penicillin, and 0.1 mg·ml^−1^ streptomycin. Parental Flp-In^TM^ T-REx^TM^ 293 cells (Invitrogen) were maintained in DMEM (high glucose) supplemented with 10% (v/v) fetal bovine serum, 2 mm l-glutamine, 100 units·ml^−1^ penicillin, 0.1 mg·ml^−1^ streptomycin, and 100 μg·ml^−1^ zeocin. Cell lines generated using Flp-In^TM^ T-REx^TM^ 293 cells as the base were maintained in DMEM (high glucose) supplemented with 10% (v/v) fetal bovine serum, 2 mm l-glutamine, 100 units·ml^−1^ penicillin, 0.1 mg·ml^−1^ streptomycin, 10 μg·ml^−1^ blasticidin, and 200 μg·ml^−1^ hygromycin.

##### Stable Cell Line Generation

Flp-In^TM^ T-REx^TM^ 293 cells were transfected with a mixture of pcDNA5/FRT/TO vector (harboring 5-HT_2C_-A206K eGFP, P-M-A206K eGFP, P-M-2xA206K eGFP, or EGFR-A206K eGFP) and the pOG44 plasmid in a 1:9 ratio with PEI (30). After 48 h, the medium was changed to medium supplemented with 200 μg·ml^−1^ hygromycin to initiate selection of stably transfected cells. Pools of cells were established (10–14 days for resistant colonies to form) and tested for inducible expression by the addition of 1 μg·ml^−1^ doxycycline for 48 h followed by screening for fluorescence corresponding to A206K eGFP and for fused A206K eGFP expression by Western blotting.

##### Generation of Cell Lysates and Western Blotting

Cells were washed once in cold PBS (120 mm NaCl, 25 mm KCl, 10 mm Na_2_HPO_4_, and 3 mm KH_2_PO_4_, pH 7.4) and harvested with ice-cold radioimmunoprecipitation assay buffer (50 mm HEPES, 150 mm NaCl, 1% Triton X-100, 0.5% sodium deoxycholate, 10 mm NaF, 5 mm EDTA, 10 mm NaH_2_PO_4_, 5% ethylene glycol, pH 7.4) supplemented with Complete protease inhibitor mixture (Roche Diagnostics, Mannheim, Germany). Extracts were passed through a 25-gauge needle and incubated for 15 min at 4 °C while on a rotating wheel. Cellular extracts were then centrifuged for 30 min at 21,000 × *g*, and the supernatant was recovered. Samples were prepared by the addition of SDS-PAGE sample buffer and heated to 65 °C for 5 min before being subjected to SDS-PAGE analysis using 4–12% BisTris gels (NuPAGE; Invitrogen) and MOPS buffer. After separation, the proteins were electrophoretically transferred to nitrocellulose membrane, which was then blocked (5% fat-free milk powder in PBS with 0.1% Tween 20) at 4 °C on a rotating shaker overnight. The membrane was incubated for 3 h with primary antibody (1:20,000 sheep anti-GFP) in 2% fat-free milk powder in PBS-Tween, washed (3 × 10 min PBS-Tween), and then incubated for 3 h with appropriate secondary antibody (horseradish peroxidase-linked rabbit anti-goat IgG, Sigma-Aldrich) diluted 1:10,000 in 2% fat-free milk powder in PBS-Tween. After washing, proteins were detected by enhanced chemiluminescence (Pierce) according to the manufacturer's instructions.

##### Cell Membrane Preparation

Cells induced with the required concentration of doxycycline to express 5-HT_2C_-A206K eGFP or other constructs were washed and then harvested with ice-cold PBS. Pellets of cells were frozen at −80 °C for a minimum of 1 h, thawed, and resuspended in ice-cold 10 mm Tris-HCl, 0.1 mm EDTA, pH 7.4 (TE buffer) supplemented with Complete protease inhibitor mixture. Cells were homogenized on ice by 40 strokes of a glass-on-Teflon homogenizer followed by centrifugation at 1000 × *g* for 5 min at 4 °C to remove unbroken cells and nuclei. The supernatant fraction was removed and passed through a 25-gauge needle 10 times before being transferred to ultracentrifuge tubes and subjected to centrifugation at 90,000 × *g* for 30 min at 4 °C. The resulting pellets were resuspended in ice-cold TE buffer. Protein concentration was assessed, and membranes were stored at −80 °C until required.

##### [^3^H]Mesulergine Binding Assays

Saturation binding curves were established by the addition of 20 μg of membrane protein to assay buffer (50 mm Tris-HCl, 100 mm NaCl, and 3 mm MgCl_2_, pH 7.5) containing varying concentrations of [^3^H]mesulergine (0.5–30 nm). Nonspecific binding was determined in the presence of 10 μm mianserin. Reactions were incubated for 60 min at 25 °C, and bound ligand was separated from free by vacuum filtration through GF/C filters (Brandel Inc., Gaithersburg, MD) that had been presoaked in 50 mm Tris-HCl, pH 7.5, 0.5% poly(ethyleneimine) (50% solution; P3143, Sigma). The filters were washed twice with cold 50 mm Tris-HCl, pH 7.5, and bound ligand was estimated by liquid scintillation spectrometry. Competition binding assays were carried out in a similar way but with a constant concentration of [^3^H]mesulergine (5 nm) and the addition of a range of concentrations of ligands of interest (0.03 nm to 10 μm). Data were analyzed using GraphPad Prism version 5.03 (GraphPad Inc.).

##### Calcium Mobilization Assays

Flp-In^TM^ T-REx^TM^ 293 cells able to express the 5-HT_2C_-A206K eGFP construct in an inducible manner were seeded into poly-d-lysine-coated, black, clear bottom 96-well microtiter plates at 50,000 cells/well for use 48 h later. 24 h after construct induction with doxycycline, the cells were loaded with the calcium-sensitive dye Fura-2, by exchanging the medium for DMEM containing 3 μm Fura-2AM. The plates were incubated in the dark for 45 min at 37 °C and then washed with 2 × 100 μl/well HEPES buffer (130 mm NaCl, 5 mm KCl, 1 mm CaCl_2_, 1 mm MgCl_2_, 20 mm HEPES, and 10 mm d-glucose, pH 7.4). 100 μl/well HEPES buffer was then added, and the plate was incubated at room temperature for 45 min in the dark. The effect of ligands was then assessed by measuring changes in Fura-2 ratio in response to calcium mobilization using a FLEX-Station (Molecular Devices, Sunnydale, CA). Data were analyzed using GraphPad Prism version 5.03.

##### Native Blue Polyacrylamide Gel Electrophoresis

Flp-In^TM^ T-REx^TM^ 293 cells induced with doxycycline to express EGFR-A206K eGFP, with or without treatment with EGF, were harvested in 1× PBS and lysed in lysis buffer (150 mm NaCl, 0.01 mm Na_3_PO_4_, 2 mm EDTA, 0.5% *n*-dodecyl β-d-maltoside, and 5% glycerol plus protease inhibitor mixture tablets, pH 7.4) on a rotating wheel for 30 min at 4 °C. Samples were then centrifuged for 30 min at 100,000 × *g* at 4 °C, and the supernatants were collected. 18 μg of solubilized supernatant plus 4 μl of G250 additive (Invitrogen) was loaded on to each lane of NativePAGE^TM^ Novex® 3–12% BisTris gels (Invitrogen). In some samples (as indicated), SDS was added 10 min prior to loading. After migration at room temperature (using buffers and conditions indicated by the manufacturer), proteins were transferred (90 min at 25 V) onto a PVDF membrane that had been prewetted for 30 s in methanol. The membrane was then fixed in 8% acetic acid for 15 min and immunoblotted with anti-GFP antiserum as described above.

##### Spatial Intensity Distribution Analysis

Flp-In^TM^ T-REx^TM^ 293 cells harboring the required A206K eGFP-tagged construct were plated down onto poly-d-lysine-coated 30-mm glass coverslips at a density of 2.5 × 10^5^ cells/coverslip. These were allowed to grow overnight and were then induced using doxycycline (concentrations as described throughout). The cells were allowed to grow overnight, and the coverslips were then rinsed in six changes of HEPES buffer (130 mm NaCl, 5 mm KCl, 1 mm CaCl_2_, 1 mm MgCl_2_, 20 mm HEPES, and 10 mm d-glucose, pH 7.4), and confocal microscope images were collected. Imaging was carried out using a Zeiss LSM 5 PASCAL EXCITER laser scanning head coupled to a Zeiss Axiovert 200M inverted microscope equipped with a ×63 plan apochromat oil immersion lens with a numerical aperture of 1.4. All samples were excited using the 488-nm line of the 25-milliwatt multiargon laser. 1024 × 1024 images were acquired using a pixel dwell time of 12.8 μs/pixel and pixel size of 0.06 or 0.09 μm. Detection was by a photomultiplier tube (PMT) using the following parameter settings: gain = 850 V, offset = 0, amplifier gain = 1. Pinhole was set to 1.00 airy unit = 96 μm. The secondary beam splitter NFT490 together with the emission filter 505LP were chosen to efficiently collect the A206K eGFP emission signal in channel 1. Laser intensity and PMT gain were chosen to minimize photobleaching and pixel saturation. To perform SpIDA analysis, it was also necessary to determine the 488-nm laser beam waist radius size, PMT shot noise, and white noise background signal. The laser beam waist radius size was estimated by imaging a *z* stack of subdiffraction-sized 100-nm Tetraspeck fluorescent microspheres (Invitrogen, catalog no. T14792). This *z* stack was imported into ImageJ, and the MetroloJ plugin was used to quantify *x*, *y*, and *z* point spread function values, which equated to 0.3156 μm for *x* and *y* and 0.6929 μm for *z*. 50 nm was removed from each of these values, and the laser beam waist area was quantified by squaring the 0.2656 μm value and multiplying it by π, which equated to a beam waist area of 0.2215 μm^2^. PMT shot noise was measured by laser spot scanning the surface of a mirror slide in the plane of focus of the microscope. The resulting reflected signal (when the 488-nm laser line was switched on) provided uniform illumination of the detector. Spot mode fluctuation signals were recorded for 30 s using a range of different laser powers until the signal saturated the PMT detector. The mean variance (standard deviation squared) of the fluctuation signals was plotted as a function of the laser intensity and the slope value calculated for the linear part of the plot. The calculated slope value defines the limit for the maximum intensity that can be analyzed using the SpIDA software. A slope value of 37.49 ± 0.081 intensity units (IU) (PMT shot noise) was applied to all image sets loaded into the SpIDA program. The white noise background signal was determined by measuring the pixel intensity values for an image acquired when the 488-nm laser line was switched off. SpIDA analysis was performed using a stand-alone MATLAB Graphical User Interface program, available at the Neurophotonics Web site (8). Images were imported into the SpIDA software, and the laser beam waist size and image pixel size were input.

The fitting of super-Poissonian distribution curves to fluorescence intensity histogram plots created from carefully selected regions of interest (RoIs) (to obtain good super-Poissonian fitting, fluorescent moieties defined within the analysis RoIs must have a homogeneous distribution) was performed on the imported input image using the SpIDA software. This forms the basis of SpIDA measurement of both quantal brightness (QB) and the number of fluorescent moieties per beam area (density) within the analyzed RoI. QB was defined as the average intensity units over time/beam waist area for each fluorescent entity investigated (monomers to higher-order oligomers). The super-Poissonian fitting procedure times varied from 1 to 5 s, depending on the population model and bin size used. The RoI sizes used contained at least 88.4–195.7 beam areas, which equates to a pixel RoI size measurement of 40 × 40 to 60 × 60. The size of the RoI plotted on each analyzed cell was dependent upon the homogeneous area available, and only one RoI was drawn on each cell analyzed. The number of cells analyzed for each experimental group ranged from 44 to 65, and number of individual images analyzed was between 15 and 20 images.

For the initial determination of the QB value of monomeric P-M-A206K eGFP, the program was used in the one-population mode, obtaining a value of (12.71 ± 0.29 IU). Subsequent measurements using the receptor-eGFP constructs used the one- or two-population fit model modes with the previously determined QB value of 12.71 IU. SpIDA monomer to dimer oligomeric analysis of the EGFR-A206K eGFP-expressing cells in response to epidermal growth factor treatment was quantified specifically using the two-population monomer-dimer model. Due to the oligomerization state complexity of the 5-HT_2C_-A206K eGFP receptor (*i.e.* initial analysis indicated that there were more than two populations present, so none of the two-population fit models could be used to yield accurate quantification), we used the one-population SpIDA model, which can accurately quantify average QB and density values in the presence of higher-order oligomeric populations.^4^ Using one-population SpIDA, the quantified QB value of monomeric P-M-A206K eGFP was used to quantify the oligomeric status of the 5-HT_2C_-A206K eGFP receptor. The SpIDA software also reports the mean fluorescent intensity for each RoI analyzed. The number of 5-HT_2C_-A206K eGFP receptors per μm^2^ (density) was measured by dividing this mean fluorescent intensity by the quantified monomeric QB value. Differences between the mean values of each experimental group data set were statistically quantified using one-way analysis of variance (GraphPad Prism version 5).

## RESULTS

### 

#### 

##### Determination of the Quantal Brightness of Membrane-localized A206K eGFP

SpIDA can be used to assess the oligomeric size of complexes of a protein tagged with an appropriate fluorophore, such as monomeric A206K eGFP, at various locations in a cell by statistical analysis of suitable laser confocal scanning images (8). We have previously expressed transiently in HEK293T cells either monomeric A206K eGFP or a tandem of this protein in which the two copies of the fluorescent polypeptide were linked by a short peptide sequence (Arg-Ile-Leu-Gln-Ser-Thr-Val-Pro-Arg-Ala-Arg-Asp-Pro-Pro-Val-Asp-Ile). RoIs within the cytoplasm where, as soluble proteins, these constructs were located were then imaged. Analysis of QB, which is proportional to the number of photons emitted by a fluorescent entity and hence to its oligomeric state, demonstrated the A206K eGFP tandem to have twice the value of the monomer (12). To generate suitable control experiments for subsequent studies on plasma membrane-associated or transmembrane proteins, we added a plasma membrane-targeting, palmitoylation-myristoylation sequence, (Met)-Gly-Cys-Ile-Asn-Ser-Lys-Arg-Lys-Asp (29), at the amino terminus of A206K eGFP and also to the tandem of A206K eGFPs. As anticipated, both following transient expression in HEK293T cells (data not shown) and following doxycycline-induced expression from the Flp-In^TM^ T-REx^TM^ locus of stably transfected Flp-In^TM^ T-REx^TM^ 293 cell lines, each of these constructs was targeted effectively to the plasma membrane (Fig. 1*A*). Such cells were treated with varying concentrations (0.25–100 ng·ml^−1^, 24 h) of doxycycline to induce expression of these constructs to different levels. Over this concentration range of the inducer, immunoblotting studies using an anti-GFP antiserum indicated substantial increases in the amount of the palmitoylation-myristoylation-modified forms of both A206K eGFP and the tandem of this fluorophore (Fig. 1*B*). Moreover, the apparent molecular mass of the predominant form of each construct, 30 kDa for the single eGFP and 60 kDa for the tandem construct, was as anticipated (Fig. 1*B*). Laser scanning confocal images of the basolateral membrane of such cells were collected (Fig. 1*C*).

**FIGURE 2.**
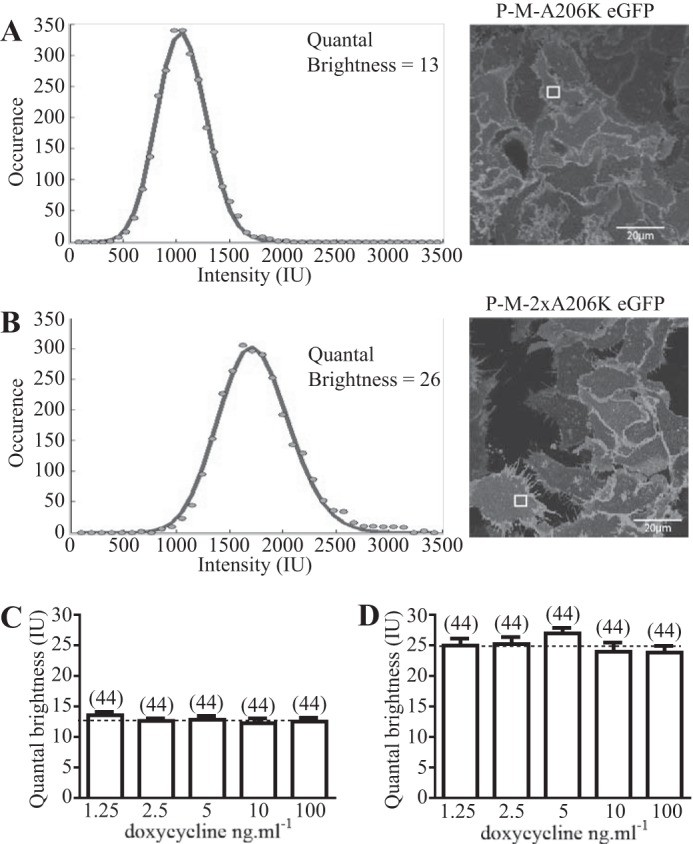
**Quantal brightness analysis of palmitoylation-myristoylation-linked forms of A206K eGFP.** QB analysis was performed on images from RoIs of the basolateral surface of cells induced to express different amounts of palmitoylation-myristoylation-linked A206K eGFP (*A*) or the palmitoylation-myristoylation-linked A206K eGFP tandem (*B*). Specific RoIs (*highlighted squares*) and their associated intensity histograms are shown for illustrative purposes. 44 distinct RoIs were analyzed for each doxycycline concentration. No difference in QB was observed for palmitoylation-myristoylation-linked A206K eGFP at different doxycycline concentrations; therefore, these values were pooled to provide QB = 12.71 ± 0.29 (mean ± S.E., *n* = 220) (*broken line*) for this construct (*C*). QB values (24.98 ± 0.53, mean ± S.E. (*error bars*), *n* = 220) were also assessed for the palmitoylation-myristoylation-linked A206K eGFP tandem at different doxycycline concentrations (*D*). Twice QB of palmitoylation-myristoylation-linked A206K eGFP is noted as the *broken line* in *D*.

Analysis of individual RoIs from cells expressing either the palmitoylation-myristoylation-linked A206K eGFP construct (Fig. 2*A*) or the palmitoylation-myristoylation-linked A206K eGFP tandem (Fig. 2*B*) demonstrated an excellent data fit to the theory. Across a range of construct expression levels, analysis of images of the RoIs within the surface of cells expressing the palmitoylation-myristoylation-linked A206K eGFP construct indicated that QB values were statistically not different (Fig. 2*C*) with, across the full data set, a value of 12.71 ± 0.29 IU (mean ± S.E., *n* = 220). Analysis of images taken from cells expressing varying amounts of the palmitoylation-myristoylation-linked A206K eGFP tandem also indicated that QB values were statistically not different across the range of expression levels achieved (Fig. 2*D*). However, now QB values across the full data set, 24.98 ± 0.53 IU (mean ± S.E., *n* = 220), were almost exactly twice (1.97 ± 0.04 times) those of the palmitoylation-myristoylation-linked single A206K eGFP construct value (Fig. 2*D*). The tandem was, therefore, identified as corresponding to a dimer.

##### Regulation of the Oligomeric State of the EGFR Can Be Determined by SpIDA

**FIGURE 3.**
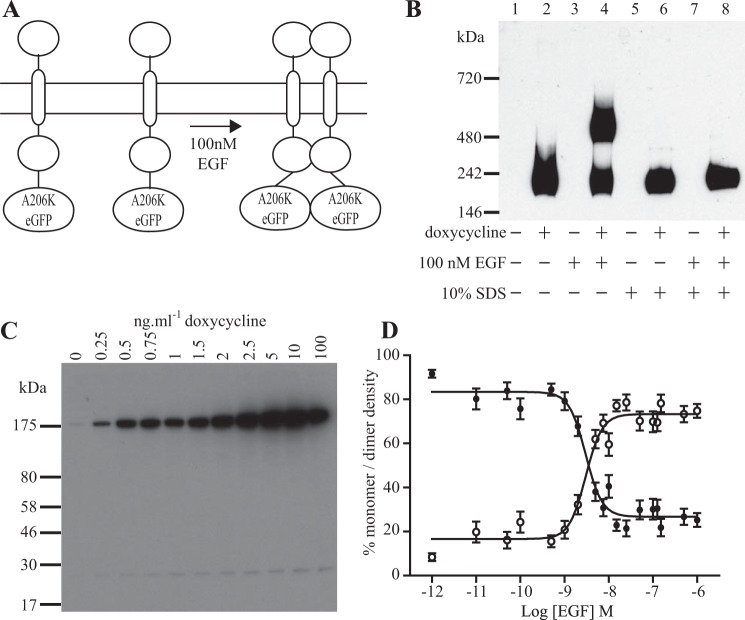
**SpIDA detects agonist-induced dimerization of EGFR-A206K eGFP.** *A*, the *schematic diagram* illustrates the conversion of monomeric EGFR-A206K eGFP into a dimeric form upon the addition of 100 nm EGF. *B*, lysates of a Flp-In^TM^ T-REx^TM^ 293 cell line induced to express EGFR-A206K eGFP (*lanes 2*, *4*, *6*, and *8*) or not induced (*lanes 1*, *3*, *5*, and *7*). Cells, either untreated (*lanes 1*, *2*, *5*, and *6*) or treated with EGF (100 nm, 10 min, 37 °C) prior to the preparation of lysate (*lanes 3*, *4*, *7*, and *8*), were then added directly to a blue native gel (*lanes 1*, *2*, *3*, and *4*); 1% (w/v) SDS was added beforehand (*lanes 5*, *6*, *7*, and *8*). Lysates were then resolved and subsequently immunoblotted with an anti-GFP antiserum. *C*, varying amounts of EGFR-A206K eGFP are produced in response to differing concentrations of doxycycline. *D*, cells induced to express EGFR-A206K eGFP were treated with varying concentrations of EGF and processed for SpIDA and QB analysis. The proportion of EGFR-A206K eGFP calculated to be present as a monomer in RoIs was greatly reduced by treatment with the ligand, whereas the proportion present as a dimer increased in parallel. *Open symbols*, proportion of EGFR-eGFP detected as dimers; *filled symbols*, proportion present as monomers. Half-maximal effect was produced with ∼3 nm EGF. *Error bars*, S.E.

To assess the capacity of SpIDA to monitor potential ligand-induced alteration in oligomeric organization of a transmembrane protein, we selected the single transmembrane pass EGFR. After the addition of A206K eGFP to the carboxyl-terminal tail of the full-length receptor, this construct was also used to generate stable Flp-In^TM^ T-REx^TM^ 293 cells in which expression of the construct was under doxycycline-inducible control. It is well established that binding of EGF promotes or stabilizes dimerization of the receptor (5) (Fig. 3*A*). To assess the extent of this, following doxycycline-induced expression of EGFR-A206K eGFP, cells were treated with or without 100 nm EGF for 10 min at 37 °C. Cell lysates were then generated and applied to non-denaturing blue native polyacrylamide gels, either with or without the preaddition of SDS to disrupt non-covalent associations. Subsequent immunoblotting demonstrated that in the absence of EGF, virtually all of the EGFR-A206K eGFP construct was monomeric (Fig. 3*B*). However, treatment with EGF resulted in a substantial proportion of the receptor construct migrating as a larger complex (Fig. 3*B*). This was not a reflection of a covalent modification because the addition of SDS to such samples prior to resolution on the blue native polyacrylamide gel resulted in all of the receptor migrating as a monomer (Fig. 3*B*). As for the palmitoylation-myristoylation motif-linked form of A206K eGFP, varying amounts of EGFR-A206K eGFP were present in the stable Flp-In^TM^ T-REx^TM^ 293 cells after treatment with differing concentrations of doxycycline (Fig. 3*C*). SpIDA analysis of EGFR-A206K eGFP organization in the basolateral membrane indicated that in the absence of EGF, the EGFR-A206K eGFP construct was present largely as a monomer (Fig. 3*D*). The addition of 100 nm EGF resulted in rapid interactions, such that within 10 min, some 75% of the construct was now dimeric (Fig. 3*D*). Application of a range of concentrations of EGF demonstrated that this conversion from monomer to dimer was concentration-dependent with EC_50_ = 2.99 ± 0.07 nm (Fig. 3*D*).

##### The Human 5-HT_2C_ Receptor Labeled at Its Carboxyl Terminus with A206K eGFP Is Functional and Able to Bind a Selective Antagonist with High Affinity

**FIGURE 4.**
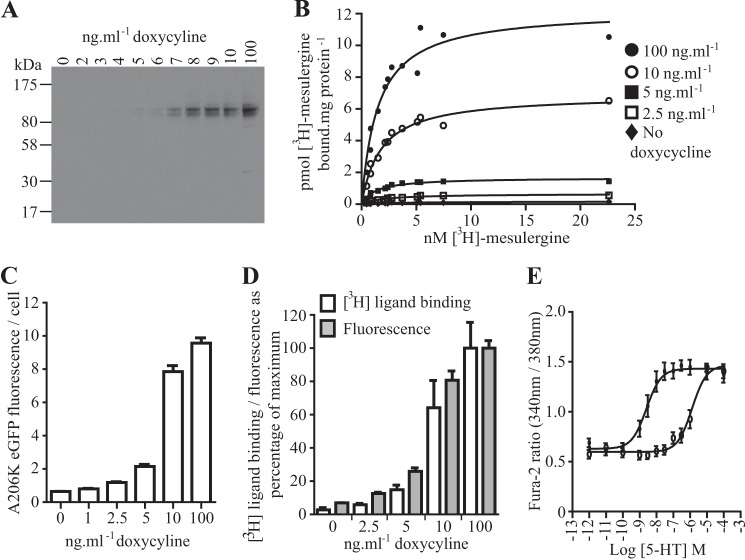
**Characterization of a Flp-In^TM^ T-REx^TM^ 293 cell line able to express 5-HT_2C_-A206K eGFP.** A Flp-In^TM^ T-REx^TM^ 293 cell line stably harboring 5-HT_2C_-A206K eGFP was generated. Treatment of these cells with varying concentrations of doxycycline resulted in differing levels of expression of the receptor construct as assessed by immunoblotting of SDS-PAGE-resolved cell lysates with an anti-GFP antiserum (*A*), the specific binding of the antagonist ligand [^3^H]mersulergine (*symbols* correspond to the indicated concentration of doxycycline used to induce construct expression) (*B*), and detection of A206K eGFP fluorescence emission (*C*). These two measures of construct expression level were closely correlated (*D*). This construct was functional. *E*, 5-HT stimulated a concentration-dependent increase of [Ca^2+^]*_i_* in doxycycline-induced cells (*closed circles*) that was antagonized by the 5-HT_2C_-selective blocker SB242084 (10 μm) (*open circles*). *Error bars*, S.E.

The serotonin 5-HT_2C_ receptor is a well studied member of the family of rhodopsin-like GPCRs (31) and is the molecular target of lorcaserin HCl, recently approved by the Federal Drug Administration as BELVIQ, a medicine for chronic weight management (32). The 5-HT_2C_ receptor has been described, based on analysis of data obtained from fluorescence correlation spectroscopy (FCS), to exist as a strict dimer at various expression levels (33). We expressed human 5-HT_2C_-A206K eGFP stably at the Flp-In^TM^ T-REx^TM^ locus of Flp-In^TM^ T-REx^TM^ 293 cells and induced varying levels of this construct by treatment with a range of concentrations of doxycycline (Fig. 4). This was assessed qualitatively via immunoblotting studies (Fig. 4*A*) and quantified by measuring either the specific binding of [^3^H]mesulergine, a high affinity antagonist of the 5-HT_2C_ receptor (Fig. 4*B*), or simply by measuring fluorescence emission of A206K GFP (Fig. 4*C*). These two parameters provided equivalent information on relative and absolute expression levels because they were highly correlated (Fig. 4*D*). Expressed 5-HT_2C_-A206K eGFP was functional. The addition of 5-HT to cells induced to express the receptor construct resulted in a large, concentration-dependent, increase in [Ca^2+^]*_i_* that was antagonized, in a surmountable manner, by the co-addition of the highly selective 5-HT_2C_ receptor antagonist SB242084 (Fig. 4*E*).

##### The Oligomeric State of the 5HT_2C_ Receptor Analyzed by SpIDA

**FIGURE 5.**
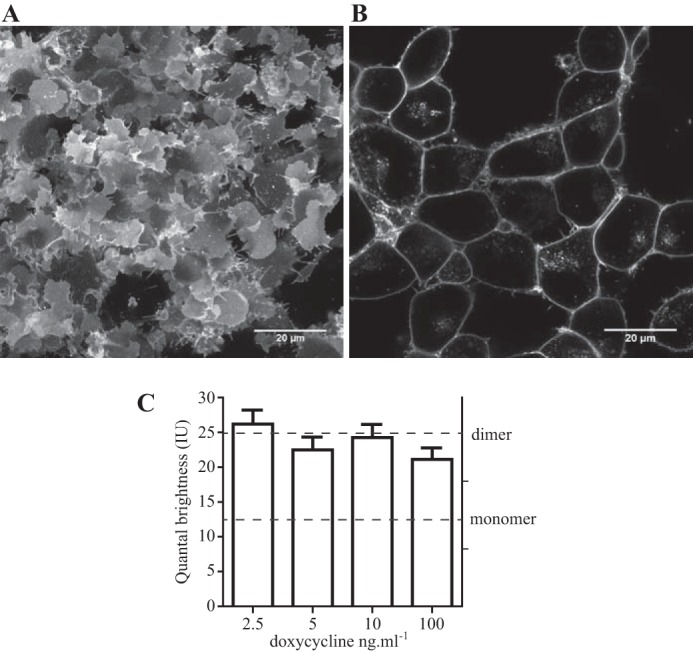
**Initial analysis of quantal brightness of 5-HT_2C_-A206K eGFP suggests it to be a constitutive dimer.** Induction of expression of 5-HT_2C_-A206K eGFP from the Flp-In^TM^ T-REx^TM^ locus of cells harboring this construct resulted in cell surface delivery. *A*, basolateral image; *B*, confocal image across the center of a group of cells. *Scale bars*, 20 μm. QB analysis performed after induction of 5-HT_2C_-A206K eGFP with varying concentrations of doxycycline produced data consistent with the receptor being predominantly dimeric (*C*). *Error bars*, S.E.

**FIGURE 6.**
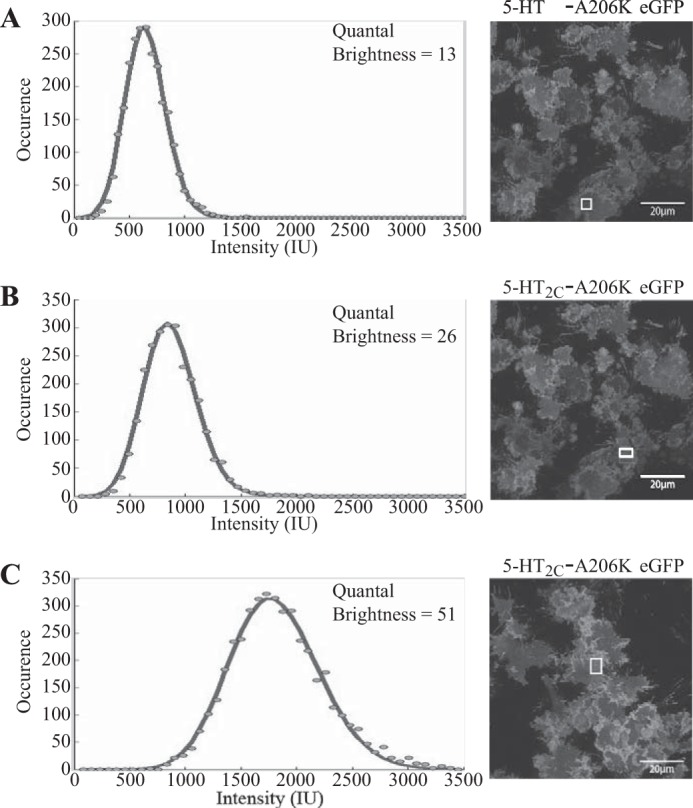
**Quantal brightness analysis of 5-HT_2C_-A206K eGFP.** QB analysis was performed on images from RoI of the basolateral surface of cells induced to express 5-HT_2C_-A206K eGFP. Specific RoI (*highlighted*) within the confocal images and their associated intensity histograms are shown for illustrative purposes (*A–C*). Each intensity histogram was fit to a single population model with results consistent with the receptor being a monomer (*A*), a dimer (*B*), and a tetramer (*C*).

**FIGURE 7.**
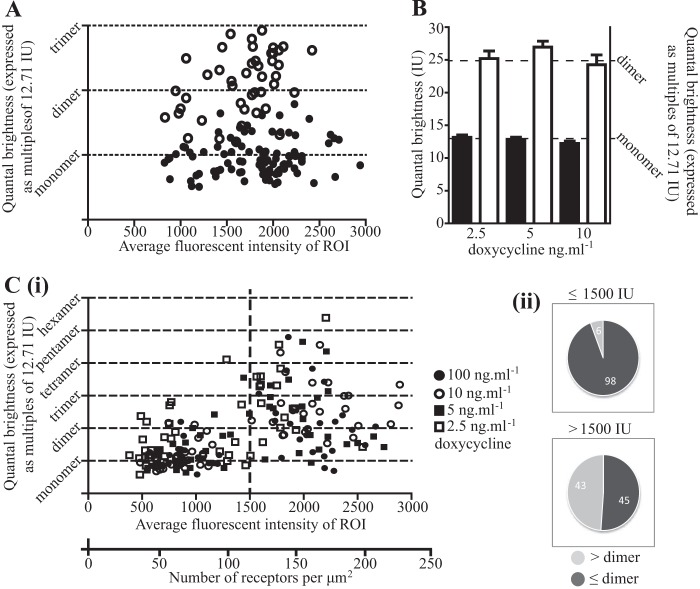
**Analysis of quantal brightness in RoI with varying fluorescence intensity.** Quantal brightness data generated from cells expressing the palmitoylation-myristoylation-linked A206K eGFP (*filled symbols*) and palmitoylation-myristoylation-linked A206K eGFP tandem (*open symbols*) are plotted against the average fluorescence intensity value for each RoI examined (*A*). Values for the single A206K eGFP cluster around the monomer *dashed line* across the fluorescence intensity scale, and those for the A206K eGFP tandem cluster around the *dimer line*. Mean ± S.E. (*error bars*) of QB for cells induced with varying concentrations of doxycycline are shown in *B. Filled bars*, palmitoylation-myristoylation-linked A206K eGFP; *open bars*, palmitoylation-myristoylation-linked A206K eGFP tandem. By contrast, analysis in the manner shown in *A* of QB values of 5-HT_2C_-A206K eGFP indicates that in regions of low average fluorescence intensity, the construct is predominantly a monomer, but in RoIs of higher fluorescence intensity the receptor was predominantly a series of dimers and higher-order oligomers (*C*). This is seen over a wide range of doxycycline concentrations and hence expression levels (*closed circles*, 100 ng·ml^−1^ doxycycline; *open circles*, 10 ng·ml^−1^ doxycycline; *closed squares*, 5 ng·ml^−1^ doxycycline; *open squares* 2.5 ng·ml^−1^ doxycycline (*i*). Using an arbitrary cut-off, 48.9% (43 of 88) of QB values for 5-HT_2C_-A206K eGFP were greater than mean dimer + 3 × S.E. at fluorescence intensity values greater than 1500, whereas at fluorescence intensity values less than 1500, this was 5.7% (6 of 104) (*ii*).

As anticipated for a transmembrane receptor, 5-HT_2C_-A206K eGFP was also effectively delivered to the cell surface when expression was induced in this cell line (Fig. 5, *A* and *B*). QB analysis of 5-HT_2C_-A206K eGFP, 23.52 ± 0.92 IU (mean ± S.E., *n* = 192), did not vary statistically across a range of expression levels (Fig. 5*C*), and the average QB of 5-HT_2C_-A206K eGFP across the full data set was close to twice (1.85 ± 0.21 times, mean ± S.E., *n* = 192) that of the palmitoylation-myristoylation-linked single A206K eGFP (Fig. 5*C*). These data would be consistent, as argued previously (33), with the 5-HT_2C_-A206K eGFP construct being, predominantly, a constitutive dimer over a range of expression levels. However, QB in different RoIs expressing 5-HT_2C_-A206K eGFP displayed a substantial range, although the data fit for individual RoIs was routinely excellent (Fig. 6). This suggested a more complex pattern than for the palmitoylation-myristoylation-linked forms of A206K eGFP. In RoIs from cells expressing the 5-HT_2C_-A206K eGFP construct, and in cells expressing the palmitoylation-myristoylation-linked forms of A206K eGFP, visual inspection and quantification of the images showed a wide range of fluorescence intensity values across the RoIs examined. To explore this more fully, we analyzed initially the measured QB values for each RoI taken from cells expressing the palmitoylation-myristoylation-linked single A206K eGFP and plotted these individually. Even following treatment with a single concentration of doxycycline, which, conceptually, should result in equivalent expression of the construct in each cell, a wide range of fluorescence intensity values were measured for different RoIs (Fig. 7*A*). Despite this variation in fluorescence intensity values, the average QB value did not vary and was consistent with palmitoylation-myristoylation-linked single A206K eGFP being a monomer in all locations and across the range of expression levels achieved (Fig. 7*B*). Similar analysis of the data from the palmitoylation-myristoylation-linked A206K eGFP tandem showed once again across the range of fluorescence intensity values measured in different RoIs that this construct was identified as dimeric (Fig. 7, *A* and *B*). In contrast, detailed analysis of cells and RoIs expressing the 5-HT_2C_-A206K eGFP construct showed a very different pattern (Fig. 7*C*). Here, when plotting QB from RoIs of varying fluorescence intensity, the predominant signal varied substantially. In regions of lower average fluorescence intensity, QB values were consistent with the receptor being predominantly monomeric with some indication of a proportion of dimers (Fig. 7*C*). However, within RoIs of substantially higher average fluorescence intensity, the QB signals were largely consistent with dimers and oligomeric entities (Fig. 7*C*). Quantification, in which QB values greater than mean dimer + 3 × S.E. values were considered to represent oligomeric forms, indicated that in RoIs with average fluorescent intensity ≤1500, the percentage of oligomers was 5.8%, whereas in RoIs with average fluorescent intensity >1500, the proportion of oligomeric forms was markedly higher at 48.9% (Fig. 7*C*).

##### The Effect of Antagonist Treatment upon the Oligomeric State of 5-HT_2C_

**FIGURE 8.**
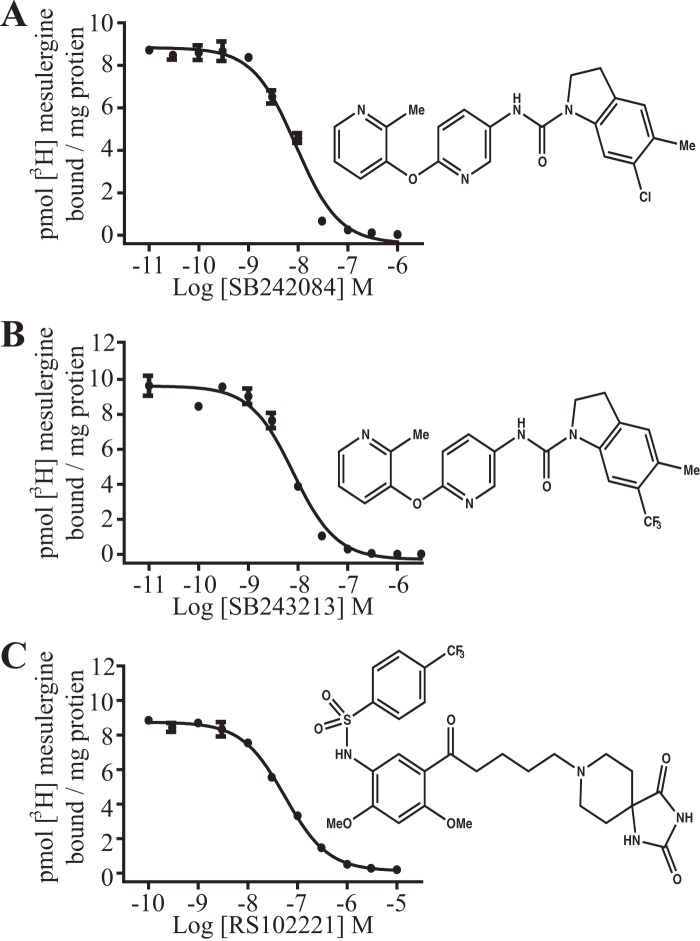
**Selective 5-HT_2C_ antagonists compete fully with [^3^H]mesulergine to bind to 5-HT_2C_-A206K eGFP and with high affinity.** Competition binding assays were performed with [^3^H]mesulergine on membranes of Flp-In^TM^ T-REx^TM^ cells induced to express 5-HT_2C_-A206K eGFP to determine the affinity of the 5-HT_2C_ selective antagonists SB242084 (*A*), SB243213 (*B*), and RS102221 (*C*) (representative data of *n* = 3 in each case). *K_i_* values were determined as follows: SB242084, 2.3 nm; SB243213, 2.1 nm; RS102221, 16.6 nm. *Error bars*, S.E.

**FIGURE 9.**
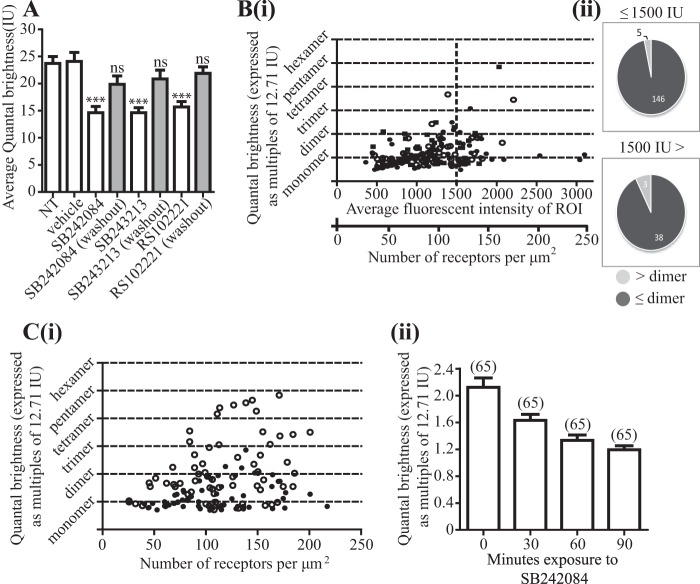
**Selective 5-HT_2C_ antagonists reduce the oligomeric organization of 5-HT_2C_-A206K eGFP and this is reversed upon washout.** Quantal brightness data were obtained from RoIs of cells expressing 5-HT_2C_-A206K eGFP that were either not treated (*NT*) or treated with vehicle or with SB242084 (50 nm), SB243213 (50 nm), or RS102221 (100 nm) for 90 min (*open bars*). Equivalent experiments were performed after washout of the ligands and a 60-min recovery period (*filled bars*). Pooled data represent means ± S.E. (*error bars*) (*A*). ***, *p* < 0.0001 *versus* nontreated; *ns*, not significantly different. *B*, complete data set for RoI of varying fluorescence intensity from cells treated with antagonists (*filled circles*, SB242084; *open circles*, SB243213; *filled squares*, RS102221) (*i*). Each antagonist/inverse antagonist produced an equivalent response. Data for the three antagonists were therefore combined. QB values for 5-HT_2C_-A206K eGFP greater than the mean of dimer + 3 × S.E. indicated that 4.2% (8 of 192) of the receptors were now oligomeric (*ii*). *C*, the effect of SB242084 was time-dependent. QB was assessed at various times before and after exposure to 50 nm SB242084. *i*, before ligand treatment (*open symbols*) or after treatment with SB242084 (90 min) (*filled symbols*). *ii*, mean ± S.E. (*error bars*) QB at various times after treatment.

We finally explored potential effects of ligands that act as antagonists/inverse agonists of the 5-HT_2C_ receptor, including SB242084, on receptor quaternary organization. In ligand binding studies performed on membranes generated from Flp-In^TM^ T-REx^TM^ 293 cells induced to express 5-HT_2C_-A206K eGFP, SB242084 was able to compete fully with [^3^H]mesulergine to bind to the receptor construct (Fig. 8*A*), with *K_i_* estimated as 2.3 nm. Two further 5-HT_2C_ receptor blockers, the highly structurally related antagonist SB243213 (*K_i_* = 2.1 nm) and RS102221 (*K_i_* = 16.6 nm), from a very distinct chemical series, were also able to fully outcompete [^3^H]mesulergine to bind the receptor construct (Fig. 8, *B* and *C*). Treatment of cells expressing 5-HT_2C_-A206K eGFP with SB242084 (50 nm, 90 min, 37 °C) before imaging of the basolateral membrane and SpIDA analysis resulted in a marked alteration in measured QB values. There was a large, statistically highly significant, reduction in average QB of 5-HT_2C_ A206K eGFP, to 1.46 ± 0.12-fold (mean ± S.E., *n* = 48) that of the palmitoylation-myristoylation-linked single A206K eGFP construct value (Fig. 9*A*). Moreover, equivalent treatment of cells with either SB243213 (50 nm) or RS102221 (100 nm) also resulted in very similar outcomes (Fig. 9*A*). Fluorescence intensity of the RoI is directly related to the number of molecules of the fluorophore imaged. Detailed analysis of QB values with fluorescence intensity of individual RoIs was consistent with the predominant form of antagonist-bound 5-HT_2C_-A206K eGFP being a monomer (Fig. 9*B*), and once more, this feature was most marked in RoIs of lower fluorescence intensity (Fig. 9*B*). Based on QB values greater than dimer + 3 × S.E. in RoIs with average fluorescent intensity ≤1500, now only 3.3% of 5-HT_2C_ A206K eGFP was oligomeric. Furthermore, even in RoIs with average fluorescent intensity >1500, the proportion of oligomeric species was only 7.3% (Fig. 9*B*). The effect of SB242084 was time-dependent (Fig. 9*C*), with half-maximal effect produced with 30 min (Fig. 9*C*). To examine the reversibility of this effect, cells expressing 5-HT_2C_-A206K eGFP were treated with SB242084 (50 nm), SB243213 (50 nm), or RS102221 (100 nm) for 90 min, and the antagonist-containing medium was then removed. The cells were washed six times in growth medium and incubated for 60 min at 37 °C. Imaging of the basolateral membrane and SpIDA analysis of these images revealed a recovery of the average QB values for 5-HT_2C_-A206K eGFP to a level not significantly different from the untreated cells (Fig. 9*A*).

## DISCUSSION

Analysis of the quaternary organization of GPCRs within the predominant rhodopsin-like or class A grouping has employed a wide range of approaches (34, 35). Despite many years of study, conclusions remain both uncertain and contentious. These range from views that such receptors are predominantly monomeric and have little potential to interact in more than a fleeting fashion (17–20) to those that posit that the receptors are predominantly dimeric (22–23) or even tetrameric (3, 24) and that such organizational structure is relatively stable. In an attempt to address this issue, herein we have employed SpIDA. SpIDA is a biophysical technique in which pixel-integrated fluorescence intensity histograms are generated from RoIs defined from confocal laser scanning microscopy images of cells expressing fluorophore-tagged or modified proteins (8–9). Histograms of the total number of pixels for each integrated fluorescence intensity value, within the selected RoI excited by the laser, are constructed and analyzed by super-Poissonian distribution functions to obtain density maps and QB values of the fluorophore-tagged protein. The distribution of the histogram provides information about the number and oligomerization state(s) of the tagged protein (8, 9).

Preliminary data for subsequent analysis of the oligomerization state of the 5-HT_2C_ receptor were provided by two separate studies. In the first, we used a co-translational/post-translational palmitoylation-myristoylation sequence from the Lyn non-receptor tyrosine kinase to anchor either a single molecule of A206K eGFP or a tandem of this protein directly to the plasma membrane of cells. These constructs were expressed from the doxycycline-regulated Flp-In^TM^ T-REx^TM^ locus of stably transfected Flp-In^TM^ T-REx^TM^ 293 cells to allow varying amounts of each form to be generated in a controlled fashion. Importantly, across expression levels, the average QB of palmitoylation-myristoylation-linked A206K eGFP was unchanged, whereas the QB of the palmitoylation-myristoylation-linked A206K eGFP tandem, although also unchanged at different expression levels, was almost exactly double. As such, two linked molecules of A206K eGFP attached to the plasma membrane were identified and quantified as a dimer. Equally importantly, when measuring the QB of these two constructs across RoIs in which the average fluorescent intensity varied considerably, the average QB values for these two constructs remained constant. This implies that even in regions of high fluorescence intensity and at the highest expression levels produced, there was no indication of “crowding” or “bystander” effects that generated artifactual dimers or higher-order complexes. Second, we took advantage of the well appreciated capacity of many single transmembrane span receptor tyrosine kinases to dimerize upon the addition of an agonist ligand to demonstrate the capacity of SpIDA to detect and quantify such changes. The addition of EGF to cells induced to express EGFR-A206K eGFP resulted in SpIDA analyses that indicated a transition from ∼90% monomer/10% dimer to 20% monomer/80% dimer. Moreover, these results were similar to those observed by resolving lysates of EGF-treated and untreated cells on non-denaturing blue native gels. Because we had previously used SpIDA to demonstrate that the single transmembrane domain axonal guidance receptor Robo-1 is a constitutive dimer (12), we confirmed SpIDA as a valid approach to assess the quaternary organization of a variety of transmembrane proteins, including, potentially, seven-transmembrane domain GPCRs.

For these studies, we selected the 5-HT_2C_ receptor. This selection was based on previous work that employed confocal FRET acceptor bleaching and FCS in combination with photon counting histogram analysis to conclude that this receptor is a strict dimer across a broad range of expression levels (33, 36). Averaging of QB values across all of the RoIs examined on cells induced to express 5-HT_2C_-A206K eGFP was generally consistent with the concept of this receptor being a constitutive dimer. However, unlike the situation with either the palmitoylation-myristoylation-linked single A206K or the palmitoylation-myristoylation-linked A206K eGFP tandem where QB and, therefore, estimated organization stoichiometry did not vary significantly with the fluorescence intensity of the RoI studied, this did vary markedly for the 5-HT_2C_-A206K eGFP construct. Although the average QB across the full data set could be interpreted as the receptor being present entirely as constitutive dimers, this conclusion was invalid and was potentially much more complex when analyzed selectively in RoIs of differing intensity. In RoIs of modest average fluorescence intensity, QB values of 5-HT_2C_-A206K eGFP were consistent with the receptor being predominantly monomeric, with a small proportion of dimers, whereas in RoIs of higher average fluorescence intensity, the proportion of monomers was much reduced, and the proportion of dimers and higher-order complexes was substantial. It is interesting to compare these results and conclusions with studies from Calebiro *et al.* (37) on the quaternary structure of the β_1_- and β_2_-adrenoceptors. Here, using a transient transfection approach, incorporation of fluorophores into SNAP-tagged (38) forms of these receptors, and single molecule tracking, these workers concluded that the number and proportion of receptor oligomers increased with expression level and that at similar expression levels, the β_2_-adrenoceptor was more likely to be oligomeric than the β_1_-adrenoceptor (37). These results are similar to the mixture of monomers, dimers, and higher-order forms of the 5-HT_2C_ receptor observed herein. Moreover, although a number of studies based on single molecule tracking support the concept that class A GPCRs can interact, in a number of cases, such interactions appear to be short term and dynamic (17, 39) rather than fixed and static (22, 23).

Previous work on the oligomerization state of the 5-HT_2C_ receptor by Herrick-Davis and colleagues (22, 23, 33) has centered on using repetitive, single illumination FCS spot measurements. FCS is particularly well suited for studying fluorescent molecules, which freely diffuse via Brownian motion in a uniform fashion through space and time within the detection volume (*i.e.* isotropic diffusion). Different sized receptor oligomer species slowly diffuse at varying rates (0.1–1 μm^2^/s), so this approach is potentially less well suited for detecting receptor oligomer species that move at varying rates in space over time within the detection volume (*i.e.* anisotropic diffusion). A further potential concern with analysis of FCS is photobleaching and cell movement. Photobleaching is common for fluorescent membrane proteins, due to their two-dimensional confinement within the plasma membrane, even if the laser intensity has been minimized. Initial FCS spot scan measurements are particularly prone to photobleaching (40). This is a particular issue with the initial scan, as noted by Herrick Davis *et al.* (33). Therefore, in their studies, the average molecular brightness was quantified using only observation spot scans 2–10. This initial apparent bleaching and its subsidence can be attributed to complete bleaching of slow moving/immobile oligomeric protein species (40) and may limit the ability of FCS to observe such slow moving/immobile oligomeric protein species. This would make them invisible during subsequent scan acquisition. This may contribute to the lack of detection of higher-order complexes reported in previous work (22, 33). In part, SpIDA was developed to overcome the FCS detection limitation issues described above. SpIDA is very well suited for detecting receptor oligomer species, which are immobile or move slowly at varying diffusion rates, and analysis via SpIDA is potentially more informative and accurate than FCS. Furthermore, SpIDA requires only a single input image acquired at a 12.8-μs dwell time/pixel on the collected image frame to quantify molecular brightness, so underestimation of molecular brightness due to photobleaching is negligible.

It was particularly interesting in the current studies that treatment of cells expressing 5-HT_2C_-A206K eGFP with ligands that are antagonists/inverse agonists at this receptor resulted in a marked reduction in the overall organizational size of the receptor population. Treatment with three distinct antagonists produced data consistent with the receptor being predominantly monomeric in the presence of these ligands. The issue of whether class A GPCR quaternary structure is altered in the presence of ligands has been as controversial as the overarching questions on their quaternary organization in the basal state (27, 41). At least for the 5-HT_2C_ receptor, the current results provide a clear answer for a group of antagonists. This is also of interest in terms of approach because a number of efforts to employ single molecule tracking of GPCRs to define quaternary organization, particularly for muscarinic acetylcholine receptor subtypes, have used fluorescently labeled antagonist ligands to label the receptor (17, 42). This relies on the basic assumptions that the ligands bind the receptors with a defined 1:1 stoichiometry and that ligand binding does not inherently alter quaternary structure. Although this now needs to be explored for other members of the class A GPCR family, including the muscarinic receptor subfamily, it is clearly not the case for the 5-HT_2C_ receptor.

In an attempt to define the basis for homomeric 5-HT_2C_ receptor interactions, Mancia *et al.* (43) introduced cysteine residues into a variety of locations within the transmembrane domains of the rat 5-HT_2C_ receptor. These variants were then employed in cross-linking studies. Interactions were observed that implicated residues in transmembrane domain I in forming a dimeric interaction. Moreover, a second potential interface involving residues within transmembrane domains IV and V was also identified. These results suggest that either there is more than one way to produce a class A receptor dimer (44) or that, as shown recently for other receptors, including the α_1b_-adrenoceptor (45) and the muscarinic M_3_ acetylcholine receptor (3, 46), higher-order oligomers can form. Future studies will consider the quaternary organization of a number of the mutants generated by (43).

To date, studies on GPCR organization have been largely restricted to the use of transfected cell systems. Although it has been possible to predict the presence of certain heteromeric GPCR complexes directly *in vivo* via analysis of behavioral phenotypes of mouse knock-out models and their recovery following replacement with, for example, a virally delivered construct (47), equivalent studies are not practical for homomeric complexes. However, studies employing either a chemical biology approach (48) or homogeneous time-resolved FRET-based labeling of receptor agonists and antagonists (49) have allowed *ex vivo* analysis of the presence of such receptor homomers. Although impressive, such studies have not yet been able to provide insight into the proportion of a receptor that is homomeric. Because SpIDA requires only confocal images of suitably labeled cells and tissues and is compatible with chemically fixed tissues, studies on knock-in lines of mice in which the receptor is modified with a suitable, monomeric fluorophore, such as A206K eGFP, may soon allow this issue to be addressed directly.

## Footnotes

4 A. G. Godin, unpublished observations.

## Footnotes

3 The abbreviations used are:

RET: resonance energy transfer
eGFP: enhanced green fluorescent protein
EGFR: epidermal growth factor receptor
FCS: fluorescence correlation spectroscopy
GPCR: G protein-coupled receptor
5-HT: 5-hydroxytryptamine
QB: quantal brightness
RoI: region of interest
SpIDA: spatial intensity distribution analysis
BisTris: 2-[bis(2-hydroxyethyl)amino]-2-(hydroxymethyl)propane-1,3-diol
P-M-A206K eGFP and P-M-2xA206K eGFP: palmitoylation-myristoylation-A206K eGFP and palmitoylation-myristoylation-2xA206K eGFP, respectively
PMT: photomultiplier tube
IU: intensity units.
